# Variation of stemness markers expression in tumor nodules from synchronous multi-focal hepatocellular carcinoma – an immunohistochemical study

**DOI:** 10.1186/s13000-017-0649-9

**Published:** 2017-08-01

**Authors:** Regina Cheuk-lam Lo, Carmen Oi-ning Leung, Kenneth Siu-ho Chok, Irene Oi-lin Ng

**Affiliations:** 1 Department of Pathology, The University of Hong Kong, University Pathology Building, Queen Mary Hospital, Pokfulam, Hong Kong, China; 2 Department of Surgery, The University of Hong Kong, Hong Kong, China; 3 State Key Laboratory for Liver Research, The University of Hong Kong, Hong Kong, China

**Keywords:** Stemness, Multifocal HCC, Heterogeneity

## Abstract

**Background:**

Advancing knowledge in molecular pathogenesis of hepatocellular carcinoma (HCC) opens up new horizons in the diagnostic, prognostic and therapeutic perspectives. Assessing the expression of molecular targets prior to definitive treatment is gaining importance in clinical practice. In this study, we investigated the variation in expression pattern of stemness markers in synchronous multi-focal HCC.

**Methods:**

In the first cohort, 21 liver explants with multi-focal HCC were examined for expression of stemness markers EpCAM, Sox9 and CK19 by immunohistochemistry (IHC). Expression data of 50 tumor nodules were analyzed to determine the concordance of expression among nodules in the same livers. In the second cohort, 14 tumor nodules from 6 multi-focal HCC cases proven as intra-hepatic metastasis were examined for Soc9 immunoexpression.

**Results:**

In the first cohort, thirty nodules from 16 cases expressed one or more markers, with Sox9 being most frequently expressed. Complete concordance of expression pattern for all 3 markers was observed in 6 cases. Discrepancy of staining degree was noted in 4 cases for EpCAM, 14 cases for Sox9, and 6 cases for CK19. A two-tier or three-tier difference in staining scores was noted in 5 cases for Sox9 and one case for CK19. With Sox9, identical tumor morphology in terms of Edmondson grading and growth pattern did not infer the same degree of immunoexpression; and the largest tumor nodule was not representative of highest IHC score. In the second cohort of intra-hepatic metastasis, complete concordance of Sox9 expression level was observed in 5 out of 6 cases; while the remaining case showed a 1-tier difference of positive staining.

**Conclusions:**

Our findings suggested that clonality of tumor nodules is apparently an important factor to infer immunoexpression pattern. When there is limited information to discern multiple primaries versus intra-hepatic metastasis in multi-focal HCC, discordant degree of stemness markers expression among tumor nodules was commonly observed especially for markers with higher frequency of expression. Pathological features alone do not necessarily indicate the expression pattern of the synchronous nodule and in this scenario examination of each tumor nodule is justified.

## Background

Cancer stemness has been one of the research spotlights in recent years. The relevance of stemness properties to tumor initiation, recurrence, metastasis and chemoresistance endows their significant clinical implications in hepatocellular carcinoma (HCC) [1–4]. Therefore, molecular markers conferring stemness phenotypes are appealing candidates for interrogation of the biology of HCC. A number of functional stemness markers in HCC have been identified and characterized such as CK19 [5], CD24 [6], CD133 [7], Sox9 [8] and EpCAM [9]. Translating these findings to clinical practice, assessing the expression of stemness markers in HCC samples is a potential means to define the prognosis of HCC patients and to devise regimens with targeted therapy. Expression of stemness markers in HCC was found to be associated with more aggressive biological behavior [10–13]. The stemness element in HCC has also brought about evolution of classification and nomenclature of primary liver cancer [14]. For instance, expression of CK19 in an HCC by morphology was regarded by some as “HCC with stem/progenitor cell immunophenotype” [15].

Immunohistochemical (IHC) expression of markers in tumor tissues can be heterogeneous. This also applies to cases presented with synchronous tumor nodules. HCC frequently occurs as multiple tumor nodules which may represent multi-centric tumors or intra-hepatic metastasis. In daily pathology practice, distinction between the two may not be feasible in every single case. However, in view of the foreseeable gaining popularity of liver tumor biopsy prior to treatment for delineation of gene expression profile, the potential variation of expression patterns among synchronous tumor nodules warrant further attention and clarification. In this study, we asked to what extent the expression of well-characterized stemness markers might vary among different tumor nodules in the same liver specimens. To answer this question, we made use of the liver explant model to assess the IHC expression of stemness markers EpCAM, CK19 and Sox9 in synchronous HCC tumor nodules. In order to gain further insights how clonality of the tumor nodules might affect the immunoexpression, we also included a second cohort of multi-focal HCC cases with proven evidence of intra-hepatic metastasis for comparison.

## Methods

### Clinical samples

Human tissues from the HCC cohort were obtained from patients undergoing liver transplantation at Queen Mary Hospital, Hong Kong from 2009 to 2016. All specimens collected fixed in 10% formalin for paraffin embedding. Representative tissue block(s) were taken from each tumor nodule identified on gross examination. Liver specimens with 2 to 4 tumor nodules were included in this study. Cases with tumor nodules showing extensive necrosis were excluded. Dominant nodule was defined as the tumor nodule with the greatest dimension. The second HCC cohort of intra-hepatic metastasis comprised 6 cases resected from Queen Mary Hospital, Hong Kong from 1993 to 1999. Use of clinical samples was approved by the Institutional Review Board of the University of Hong Kong/Hospital Authority Hong Kong West Cluster (Ref. UW11–424). Clinical information was retrieved from patients’ records.

### Immunohistochemistry

IHC staining for EpCAM, CK19 and Sox9 were performed on formalin-fixed, paraffin-embedded sections using labeled horseradish peroxidase (HRP) method. After heat antigen retrieval with Tris- EDTA buffer (Sox9, CK19) or Protease K enzyme antigen retrieval (EpCAM), endogenous peroxidase activities were quenched by 3% H_2_O_2_. The sections were immersed in serum free-protein block solution (Dako) and incubated with anti-Sox9 (Millipore AB5535, MA at dilution 1:1000), anti-CK19 (ab52625, Abcam) at dilution 1:1000), and anti-EpCAM (M0804, Dako at dilution 1:100) at 4°C overnight. The sections were thoroughly washed and incubated with EnvisionTM HRP-conjugated secondary antibody (Dako). Positive signals were visualized using 3,3′-diaminobenzidine (Dako). Nuclei were counterstained with hematoxylin.

### Histological assessment

The histological sections were reviewed by a liver pathologist (RCL) to confirm the diagnosis of HCC and to ascertain the viability of tumor cells. Tumor cell grading by the Edmondson grading system [16] and growth pattern of tumor cells were assessed. IHC expression results were examined with reference to a semi-quantitative method defined by the percentage of positive-staining tumor cells in the sections, with score 0 (negative): no staining; score 1: 1–33%; score 2: 34–66%; score 3: 67% or more.

## Results

### Clinicopathological parameters of the study cohort

The 21 liver explants came from 19 male patients and 2 female patients ranging from 49 to 66 years of age. All explant livers were cirrhotic, of which the etiology is in majority chronic hepatitis B virus infection (*n* = 16), followed by chronic hepatitis C virus infection (*n* = 3), alcoholic liver disease (*n* = 1) and cryptogenic (*n* = 1). Altogether 50 HCC nodules were assessed in the 21 cases. Two tumor nodules were present in 14 cases, three tumor nodules in 6, and 4 nodules in one case. The size of the tumor nodules ranged from 1 to 6.3 cm. The dimension of the dominant nodules ranged from 2 to 6.3 cm. For the Edmondson grading on tumor cell differentiation, 37 (of 50) nodules belonged to grade II and 13 belonged to grade III. Ten of the 21 cases comprised a mixed composition of grades II and III tumor nodules. Majority of tumor nodules (41 of 50) showed a trabecular growth pattern while the remaining showed a mixed trabecular and pseudoacinar pattern. The clinicopathological parameters of the 21 cases were summarized in Table 1.

**Table 1:** Clinicopathological features of the first cohort

Case no.	Gender/Age	Etiology	Nodule designation	Size of tumor nodule (mm)	Grading^a^	Growth pattern^#^
1	M/60	HBV	i ii	25 15	2 2	T T
2	M/56	HBV	i ii	35 30	2 2	T T + A
3	M/58	HBV	i ii iii iv	50 30 20 10	2 2 2 3	T + A A + T T T
4	M/59	HBV	i ii	22 18	3 2	T A + T
5	M/57	HCV	i ii	40 30	2 2	T T
6	M/51	HBV	i ii	22 10	2 2	T T
7	M/66	HBV	i ii	63 14	2 3	T T
8	M/49	HBV	i ii iii	25 20 12	2 2 3	T T T
9	M/50	HCV	i ii	40 20	2 2	T A + T
10	M/65	HBV	i ii iii	45 20 15	2 2 3	A + T T T
11	M/60	HBV	i ii	50 25	3 2	T T
12	M/55	HBV	i ii	20 10	3 2	T T
13	M/58	HBV	i ii iii	35 25 20	2 2 2	T T T
14	M/46	HBV	i ii	20 15	2 2	T T
15	M/63	ALD	i ii iii	30 25 25	3 2 3	T T T
16	M/65	HCV	i ii	45 18	3 3	T T
17	M/53	HBV	i ii iii	20 13 13	2 3 2	T + A T T
18	M/53	HBV	i ii	34 10	2 3	T + A T
19	F/60	HBV	i ii iii	40 20 10	2 2 2	T + A T T
20	M/63	HBV	i ii	45 40	2 2	T T
21	F/60	cryptogenic	i ii	50 30	2 2	T T

^a^grading by Edmondson system

#T: trabecular; A: pseudoacinar; denoted as “predominant pattern + minor pattern” in cases with mixed pattern

HBV: hepatitis B virus

HCV: hepatitis C virus

ALD: alcoholic liver disease

### Immunohistochemical expression of the stemness markers

**Fig. 1:**
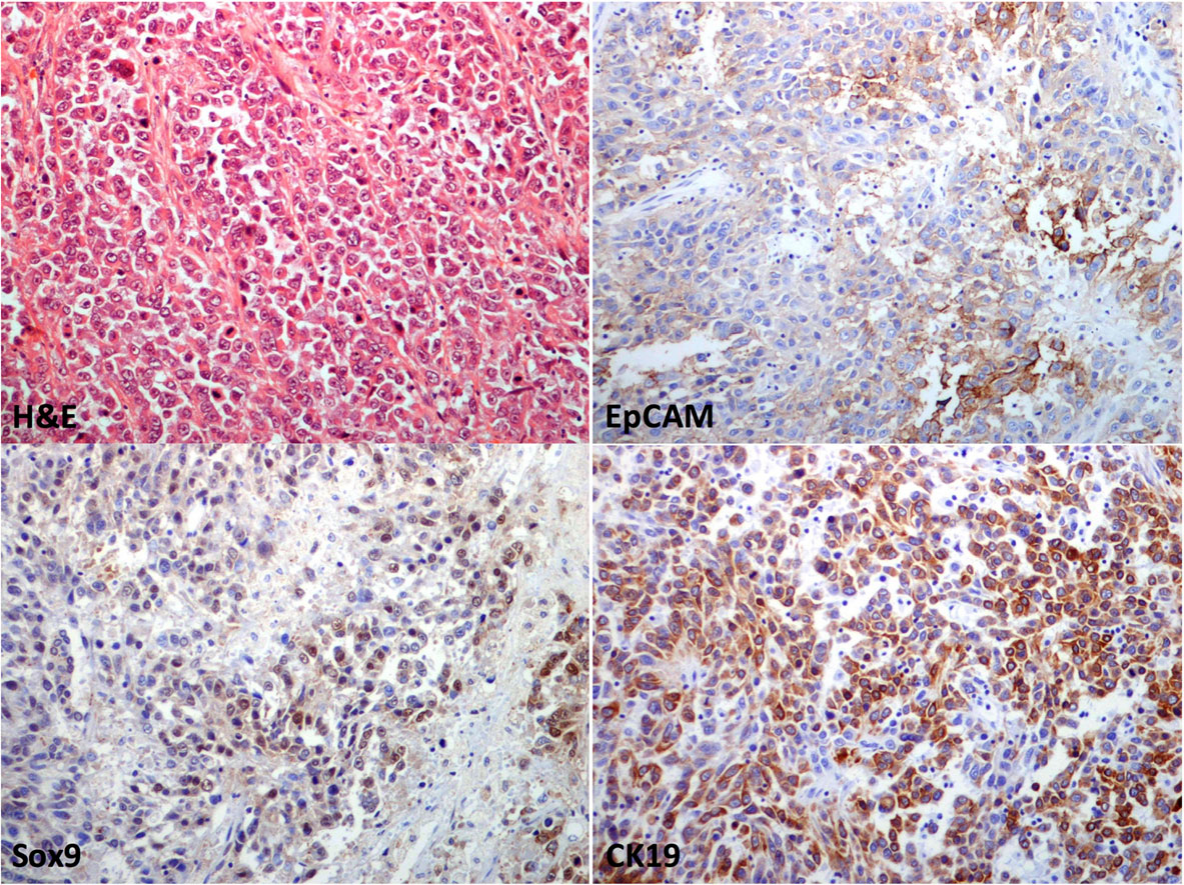
Representative images of immunohistochemical staining in the HCC tumor tissue (case#12). (A) H&E, (B) EpCAM, (C) Sox9 and (D) CK19 (×200 magnification)

The immunohistochemical expression results were presented in Table 2. EpCAM was expressed in 1 tumor nodule each from 4 cases. Sox9 was expressed in 30 nodules from 16 cases; CK19 was expressed in 6 nodules from 6 cases (Fig. 1). On the staining scores, all positive staining for EpCAM and all except one positive staining for CK19 belonged to score 1. Sox9 staining consisted of a spectrum of scores 1 to 3. Thirty nodules expressed one or more markers; 5 cases did not express any of the 3 markers in all tumor nodules.

**Table 2:** Immunohistochemical expression results of the tumor nodules in the first cohort

Case no.	Nodule designation	EpCAM^%^	Sox9^%^	CK19^%^
1	i ii	0 0	1 0	0 0
2	i ii	0 0	0 0	0 0
3	i ii iii iv	1 0 0 0	1 1 1 0	0 0 1 0
4	i ii	0 0	1 1	0 0
5	i ii	0 0	3 1	0 0
6	i ii	0 0	3 1	0 0
7	i ii	0 0	0 3	0 0
8	i ii iii	0 0 0	1 1 2	0 0 0
9	i ii	1 0	2 2	0 0
10	i ii iii	0 1 0	0 2 1	0 1 0
11	i ii	0 0	0 0	0 0
12	i ii	1 0	2 1	3 0
13	i ii iii	0 0 0	0 0 0	0 0 0
14	i ii	0 0	0 1	0 1
15	i ii iii	0 0 0	2 1 1	0 0 0
16	i ii	0 0	0 1	0 0
17	i ii iii	0 0 0	0 1 1	0 1 0
18	i ii	0 0	0 0	0 0
19	i ii iii	0 0 0	2 0 1	1 0 0
20	i ii	0 0	0 0	0 0
21	i ii	0 0	0 1	0 0

^%^score 0: no staining; score 1: 1–33%; score 2: 34–66%; score 3: >66%

### Concordance of IHC expression in multi-focal HCC

Complete concordance of expression pattern for all 3 markers was observed in 6 cases including the 5 cases showing no expression of any markers in all tumor nodules. Discrepancy of staining score was noted in 4 cases for EpCAM, 14 cases for Sox9, and 6 cases for CK19. “One-tier” discrepancy (difference by 1 grading score) was common for all 3 stains, and including all 4 cases for EpCAM and 5 cases for CK19. A two-tier or three-tier difference was noted in 5 cases for Sox9 and one case for CK19. A “positive-negative discrepancy” (i.e. 0 versus 1 or 2 or 3) for anyone stain was identified in 11 cases, with vast majority involving a “0” versus “1” difference.

Seven cases in our cohort consisted of 3 or more tumor nodules. Concordant staining score was observed in 2 cases for EpCAM, 6 for Sox9 and 6 for CK19. In 4 cases (case#3, #10, #17, #19), the scoring pattern for each of the tumor nodules was different from one another. In 2 cases (case #8, #15), two of the three tumor nodules demonstrated identical scores and in the remaining case (case#13), there was no expression for all three markers in all tumor nodules.

### Correlation of expression pattern and pathological parameters

Eleven cases showed identical tumor morphology in terms of tumor grading and growth pattern in 2 or more tumor nodules. Among the 11 cases, concordant staining score was noted in 11, 3 and 10 cases for EpCAM, Sox9 and CK19 respectively. Identical staining pattern for all 3 markers was noted in 2 cases. Lastly, we attempted to look into the potential significance of the dominant nodule, the tumor nodule of greatest dimension of each liver explant specimen. The dominant nodule corresponded to the highest score in 20 cases for EpCAM, 4 cases for Sox9 and 17 cases for CK19.

### Sox9 expression in HCC cohort of intra-hepatic metastasis

Given that the tumor nodules from multi-focal HCC could possibly represent multiple primaries or intra-hepatic metastasis, we attempted to investigate how clonality may affect the results. Since Sox9 is the most frequently expressed stemness marker from our findings above, we proceeded to examine the immunohistochemical expression of Sox9 in a cohort of 6 multi-focal HCC cases, which were proven to be intra-hepatic metastasis as determined by DNA fingerprinting using loss of heterozygosity (LOH) assay, comparative genomic hybridization (CGH) [17]. Results of 14 nodules from 6 cases were assessed and presented in Table 3. Complete concordance in staining score was observed in 5 cases. In the remaining one case, the two tumor nodules showed score 1 and score 2 respectively.

**Table 3:** Sox9 immunohistochemical expression results of the tumor nodules in the intra-hepatic metastasis cohort

Case no./Sox9 expression	nodule 1	nodule 2	nodule 3
22	2	2	2
23	1	1	1
24	3	3	n.a.
25	3	3	n.a.
26	2	1	n.a.
27	1	1	n.a.

## Discussion

In this study, we assessed and compared the immunohistochemical expression of stemness markers EpCAM, Sox9 and CK19 in HCC tissues from a cohort of liver explant cases consisting of multiple tumor nodules. Sox9 was most readily expressed in the tumor nodules the cohort, followed by CK19 and then EpCAM. The expression rates were more or less consistent with those stated in previous reports [8, 10, 11]. Complete concordance of expression pattern for all 3 markers was present in 6 cases. Of note, 5 cases showed no expression for all three markers and the concordance could be partly attributed to the low expression rates of some markers such as EpCAM and CK19. Discrepancy in expression scores was a common event, and a two-tier or three-tier discrepancy was noted in 6 (about one-fourth) cases mainly involving Sox9 staining. On the other hand, Sox9 expression in the intra-hepatic metastasis cohort revealed a high degree of concordance.

The discrepancy in IHC score among the tumor nodules within the same liver specimens could possibly be explained in part by intra-tumoral heterogeneity [18, 19]. Yet in routine pathology practice, in view of the concerns for resources and cost-effectiveness, it is impractical to perform the same IHC stain on multiple tissue blocks from the same tumor nodule. As a matter of fact, core biopsy is usually the type of specimen available for initial pathological diagnosis.

In this study, we attempted to explore whether a morphological concordance among tumor nodules could be inferring an identical pattern of stemness marker immunoexpression. While it was apparently the case for EpCAM and CK19, the results were possibly due to the relative low frequency of expression with these two markers. For Sox9 staining, an identical morphology corresponded to the same staining score in only minority of cases. Similarly, the expression of Sox9 in the dominant nodule was representative of the highest degree among all tumor nodules in the same liver specimen in only 14 of 22 cases. The above results suggested that pathological parameters alone may not be dependable for estimating the stemness markers expression of all tumor nodules in the same liver especially for markers with higher frequency of expression in HCC. Thus targeting the tumor nodule of greatest dimension only at image-guided biopsy may result in suboptimal characterization.

Liver transplantation is a best curative treatment modality for HCC. In that availability of liver grafts is always a concern and this leads to the introduction of various bridging therapies such as high-intensity focused ultrasound, trans-arterial chemoembolization and more recently stereotactic body radiotherapy [20, 21] which aim at tumor control while awaiting suitable grafts. With accumulating knowledge in the molecular pathogenesis of HCC, the spectrum of neoadjuvant therapy is anticipated to be expanding. Targeted therapy against functional molecular markers conferring stemness properties is a promising direction in this regard. In addition, recent studies indicated a potential role of stemness marker expression signature for prognostication and extension of inclusion criteria for HCC patients to be treated by liver transplantation [22]. In view of this evolving picture of clinical practice, our present study serves as a pilot investigation on the variation of well-characterized stemness markers expression in synchronous multi-focal HCC.

## Conclusion

Despite the fact that we do not have information for all the cases to determine the clonality of the tumor nodules at a molecular level such as by loss-of-heterozygosity analysis of specific DNA microsatellite loci or genomic studies [23], our study results provide some insights from a practical point of view concerning the heterogeneity of stemness markers expression among synchronous tumor nodules in HCC. When discerning between multiple primaries or intra-hepatic metastasis is limited, expression of stemness markers can be heterogeneous among tumor nodules and evaluation of one nodule is unlikely to provide insight on the expression pattern of other nodules. Our findings suggested the significance of assessing expression of all nodules at initial diagnosis. Moreover, validation works in the future would be warranted to draw conclusions for individual markers.

## Abbreviations

HCC: Hepatocellular carcinoma
IHC: Immunohistochemical

## References

[1] Jordan CT, Guzman ML, Noble M (2006). Cancer stem cells. N Engl J Med.

[2] Ma S, Lee TK, Zheng BJ, Chan KW, Guan XY (2008). CD133+ HCC cancer stem cells confer chemoresistance by preferential expression of the Akt/PKB survival pathway. Oncogene.

[3] Malanchi I, Santamaria-Martinez A, Susanto E (2011). Interactions between cancer stem cells and their niche govern metastatic colonization. Nature.

[4] Marquardt JU, Thorgeirsson SS (2010). Stem cells in hepatocarcinogenesis: evidence from genomic data. Semin Liver Dis.

[5] Kawai T, Yasuchika K, Ishii T (2015). Keratin 19, a cancer stem cell marker in human Hepatocellular carcinoma. Clin Cancer Res.

[6] Lee TK, Castilho A, Cheung VC, Tang KH, Ma S, Ng IO (2011). CD24(+) liver tumor-initiating cells drive self-renewal and tumor initiation through STAT3-mediated NANOG regulation. Cell Stem Cell.

[7] Ma S, Chan KW, Hu L (2007). Identification and characterization of tumorigenic liver cancer stem/progenitor cells. Gastroenterology.

[8] Leung C O, Mak W N, Kai A K, et al. Sox9 confers stemness properties in hepatocellular carcinoma through Frizzled-7 mediated Wnt/beta-catenin signaling. Oncotarget 2016; 7(20): 29371-29386. 10.18632/oncotarget.8835 PMC5045402 27105493

[9] Yamashita T, Budhu A, Forgues M, Wang XW (2007). Activation of hepatic stem cell marker EpCAM by Wnt-beta-catenin signaling in hepatocellular carcinoma. Cancer Res.

[10] Chan AW, Tong JH, Chan SL, Lai PB, To KF (2014). Expression of stemness markers (CD133 and EpCAM) in prognostication of hepatocellular carcinoma. Histopathology.

[11] Kim H, Choi GH, Na D C (2011). Human hepatocellular carcinomas with "Stemness"-related marker expression: keratin 19 expression and a poor prognosis. Hepatology.

[12] Kim H, Park YN (2014). Hepatocellular carcinomas expressing 'stemness'-related markers: clinicopathological characteristics. Dig Dis.

[13] Zhao Q, Zhou H, Liu Q (2016). Prognostic value of the expression of cancer stem cell-related markers CD133 and CD44 in hepatocellular carcinoma: from patients to patient-derived tumor xenograft models. Oncotarget.

[14] WHO (2010). Classication of Tumours of the digestive system.

[15] Roncalli M, Park YN, Di Tommaso L (2010). Histopathological classification of hepatocellular carcinoma. Dig Liver Dis.

[16] Edmondson HA, Steiner PE (1954). Primary carcinoma of the liver: a study of 100 cases among 48,900 necropsies. Cancer.

[17] Ng IO, Guan XY, Poon RT, Fan ST, Lee JM (2003). Determination of the molecular relationship between multiple tumour nodules in hepatocellular carcinoma differentiates multicentric origin from intrahepatic metastasis. J Pathol.

[18] Kanayama K, Imai H, Yoneda M, Hirokawa YS, Shiraishi T (2016). Significant intratumoral heterogeneity of human epidermal growth factor receptor 2 status in gastric cancer: a comparative study of immunohistochemistry, FISH, and dual-color in situ hybridization. Cancer Sci.

[19] Kurozumi S, Padilla M, Kurosumi M (2016). HER2 intratumoral heterogeneity analyses by concurrent HER2 gene and protein assessment for the prognosis of HER2 negative invasive breast cancer patients. Breast Cancer Res Treat.

[20] Chok KS, Cheung TT, Lo RC (2014). Pilot study of high-intensity focused ultrasound ablation as a bridging therapy for hepatocellular carcinoma patients wait-listed for liver transplantation. Liver Transpl.

[21] Sapisochin G, Barry A, Doherty M, et al. Stereotactic body radiotherapy versus TACE or RFA as a bridge to transplant in patients with hepatocellular carcinoma. An intention-to-treat analysis. J Hepatol. 2017;67(1):92–99. 10.1016/j.jhep.2017.02.022 28257902

[22] Miltiadous O, Sia D, Hoshida Y (2015). Progenitor cell markers predict outcome of patients with hepatocellular carcinoma beyond Milan criteria undergoing liver transplantation. J Hepatol.

[23] FEO F, PASCALE RM (2015). Multifocal hepatocellular carcinoma: intrahepatic metastasis or multicentric carcinogenesis?. Ann Transl Med.

